# From ether to ethernet: ensuring ethical policy in digital transformation of waitlist triage for cardiovascular procedures

**DOI:** 10.1038/s41746-024-01019-6

**Published:** 2024-02-29

**Authors:** Mihir A. Kelshiker, Karanjot Chhatwal, Patrik Bachtiger, Josephine Mansell, Nicholas S. Peters, Daniel B. Kramer

**Affiliations:** 1 National Heart and Lung Institute, Imperial College London, London, UK; 2 Imperial College Healthcare NHS Trust, London, UK; 3 Richard A. and Susan F. Smith Center for Outcomes Research, Beth Israel Deaconess Medical Center, Harvard Medical School, Boston, MA USA

**Keywords:** Health policy, Health services

Cardiovascular disease remains the leading cause of death in both the United Kingdom (UK) and worldwide^1^. For several coronary and valvular conditions, timely surgical or catheter-based interventions improve survival and quality of life^2,3^. Yet the UK National Health Service (NHS) faces an unprecedented backlog of over 370,000 patients (in a population of 67 million) awaiting critical cardiac procedures, leading to over 30,000 excess deaths^4^. In this *Comment*, we describe a novel strategy for digitally-supported prioritization for critical cardiovascular procedures. We then examine potential ethical concerns in this new clinical and population health pathway and evaluate programmatic features that may address these hazards while promoting effective technology adoption within the service provision for these most vulnerable patients.

## Digitally-supported Triage in the U.K. national health service

Previous approaches to NHS waiting list triage for cardiac procedures are significantly heterogenous, varying across NHS trusts and geographic locations. Providing safe cardiac waiting list triage is further complicated by the need for accessible and inclusive digital health platforms that serve large, diverse and often socioeconomically deprived populations. In the aftermath of the Covid-19 pandemic, waiting lists are the longest since the founding of the NHS, this year celebrating its 75th anniversary. In response to lengthening elective care waiting times following the Covid-19 pandemic, the Federation of Surgical Specialty Associations (FSSA) revised the UK risk prioritization framework to support systematic triage of patients. Patients added to the waiting list are categorized by their clinical team as ‘P1-P4’, where P1a indicates emergent surgical intervention within 24 hours, and P4 denotes those in whom treatment can be safely deferred for over three months. Despite these efforts, waitlists remain deeply problematic.

With no standardized monitoring protocols to systematically identify clinical deterioration on the waiting list, patients and the health system respectively incur substantial morbidity (sometimes mortality) and health economic burden from unplanned care events^5,6^. Such events propagate a negative feedback loop, precipitating unplanned procedural intervention following hospitalization – with subsequent further reduction of elective capacity and downstream impact on the waiting list. Performing emergent procedures in patients who have deteriorated (both functionally and physiologically) whilst on the waiting list is more complex, requires longer hospital admissions, and imposes poorer outcomes.

To address this unsustainable trajectory, the NHS North and South London Cardiac Operational Delivery Networks (ODN) – representing all eight tertiary cardiac centres in the UK capital – were in 2022 awarded national funding to deliver a novel, uniform, technology-enabled solution for waiting list monitoring and prioritization. One of the eight centres, Imperial College Healthcare NHS Trust (ICHNT), already delivers remote, technology-enabled care pathways using a ‘Virtual Hospital’ model, wherein non-specialist nurses action inbound clinical information from patients in accordance with standard operating procedures agreed with specialist teams. Patients in ‘virtual wards’ are supported to access a range of connected technologies including smartphone apps, wearable biosensors and handheld devices to record symptoms and physiological data.

**Fig. 1:**
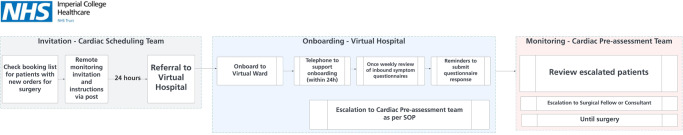
Workflow for inviting patients to participate in digitally-supported triage.

**Fig. 2:**
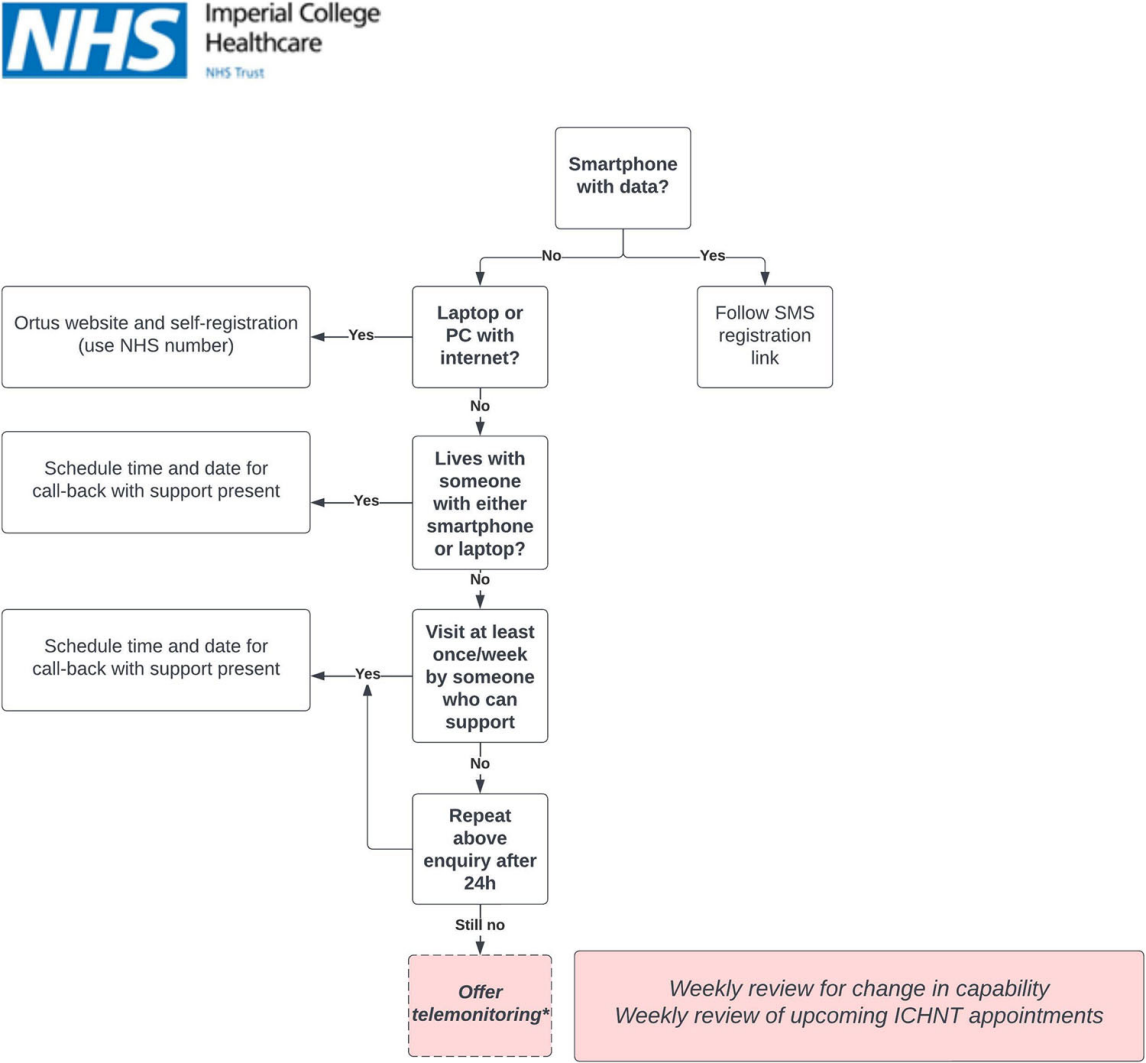
Workflow according to connectivity options.

To improve rates of detection of clinical deterioration, and reduce rates of unplanned care events and mortality, all ICHNT ‘P2’ categorized cardiac surgical patients (cardiac coronary or valvular surgery indicated within 4 weeks), in addition to all patients awaiting trans-catheter aortic valve replacement (TAVR) were offered a remote symptom monitoring pathway delivered via the Virtual Hospital (Figs. 1, 2). Patients complete weekly symptom questionnaires addressing the core clinical variables relevant to their disease state. The prioritization process occurs in three steps. First, symptom responses are automatically graded via a configurable risk categorization matrix (Supplementary Table 1). Second, the Virtual Hospital team identify those patients with a week-on-week deterioration. Finally, concerning symptoms or deterioration trigger a clinical review by the Cardiac Specialist team (by phone or in-person; see Supplementary Fig. 1). The ethical framework adopted in the triage protocol is a modified version of “sickest first”^7,8^, where self-reported symptom data is considered by specialists in the context of traditional clinical data such as objective indices of disease severity (e.g. from imaging; see Supplementary Note 1). Importantly, there is no protocolized weighting to symptom responses or objective data in the prioritization pathway, which ends with clinical judgement (no deviation from current practice). To date, over 500 patients at ICHNT, and over 4,000 patients across London have been enroled in this programme. To ensure that the programme does not defer only to utilitarian principles in trying to maximise health benefits to the greatest number of patients, our outcome measurement plan includes examining for deterioration in health status of those patients with less severe conditions.

## Policy and ethics considerations

The dire need for streamlined and standardised triage clearly justifies innovative, patient-centred solutions such as virtual wards. However, we suggest that alongside clinical outcomes for programme success (such as wait times and survival), careful attention is due towards potential ethical pitfalls. We identified key early concerns that may arise from the shift between traditional clinical triage based on bedside judgement and inpatient care to a novel, home-based, digitally-supported triage for patients with critical cardiovascular conditions awaiting definitive intervention.

### Navigation skills, equity, and gaming

The established paradigm of care often saw P2 patients waiting—days, sometimes weeks—in hospital for their procedures, particularly if clinically judged to be at ‘higher risk’. Here they would receive routine daily assessments, including vital signs (often via continuous cardiac telemetry recording of electrocardiography, and oxygenation), symptoms, and laboratory tests. These may inform patients being moved up (and down) the priority list for limited procedural slots, and may accompany general acute clinical deterioration that expedites intervention. However, patients occupying inpatient beds in this fashion may not be considered an efficient use of vitally scarce resource, particularly as the clinical evidence base for doing so is lacking, and the principal drivers include clinician risk aversion. In digitally-supported triage, patients are managed remotely, from home. Assessments are moved into an electronic platform requiring active engagement from patients. They are registered and onboarded into the system with assistance, but require at least weekly investment in reporting their own symptoms, potentially uploading digital physiological data such as heart rate, blood pressure or blood glucose, and communicating new findings to their care team to maintain or advance their place in the virtual queue.

This model shifts from traditional triage at the bedside to one where patients must navigate an electronic portal on their own devices, their own cellular or Wi-Fi network, with their own connected peripherals such as Bluetooth-enabled weight scales or electrocardiographic tracings. While any healthcare encounter involves variable navigation skills or personalized networking, digitally-supported triage makes this even more stark by making digital literacy a key factor in the patient experience. Certain patient groups (e.g. younger) may navigate smartphone applications and remote monitoring devices more nimbly, whilst other patients may feel less confident and require further guidance. The established ‘digital divide’ in healthcare also mirrors the socioeconomic gradient for health, whereby those at greatest need are often the most seldom listened to, whilst likely having poorer access to smartphones and internet connectivity.

Delivering systematic, rule-based approaches to digital inclusion is critical to ensure that nobody is left behind, whilst also maximising efficiency of limited resource. For example, telephone-based monitoring engages almost all patients, but is generally unsustainable or scalable for healthcare organisations. Therefore, we argue that if digital triage models are to be adopted by patients as the standardised model (rather than an opt-in/opt-out structure), it is imperative to identify the ‘digitally unengaged but engageable’ patient population; and maximise their practical capability, opportunity, and motivation^9^ to engage with digital platforms, such that telephone-based resource is preserved for those in greatest need who are truly digitally ‘unengageable’. Our implementation with 509 patients at ICHNT since January 2023 has achieved a 79% activation rate with the digital platform (402 patients completed app enrolment), with remaining patients receiving telephone-based monitoring. A sampling audit of 22 out of 36 patients who did not initially engage with the digital platform revealed that 6 (27%) subsequently did so after encouragement to seek support from household members or a regular visitor.

Shifting the daily objective review of signs and symptoms in large part to more subjective patient reporting also exposes triage decisions to potential risk of gamification. For example, in patients with critical aortic stenosis or severe coronary artery disease, the occurrence of chest pain with minimal activity or at rest would typically be considered indications for urgent or emergent intervention. Is it too cynical to imagine patients, families, or physicians learning (and perhaps reporting via social media or other means) what kinds of reported signs and symptoms appear to prompt brisk treatment? We observe that these are patients who, by definition, linger in difficult, tenuous circumstances with limited ability to leave their homes, potentially impacting loved ones or carers just for physical care, alongside emotional toil. Viewed in this desperate context, we believe it is worth at least considering the possibility that the system may be subject to being gamed. For example, updated triage rules amongst U.S. cardiac transplantation patients may have changed clinical decision-making regarding mechanical support or application of “exceptions” out of proportion to patient needs^10^. Digitally-supported triage, therefore, must include careful governance and oversight to detect (for example) implausible or suspicious survey responses or other data irregularities that may indicate conscious or subconscious flexing of rules that may attenuate the population impact of the programme.

### Too much of a good thing?

Patients in hospital awaiting procedures via traditional triage remain at risk of new organic or iatrogenic complications that might delay their procedures, or change their candidacy for intervention entirely. For example, a new infection requiring antibiotics, or a blood clot requiring systemic anticoagulation, may interfere with traditional triage insofar as either may preclude invasive therapy. Patients supported virtually may be at risk for these same problems, though they may be more difficult to recognize and treat, and potentially also new findings *exclusive* to digitally-supported triage. For example, consider inclusion in a digital portal data gleaned from selected sleep monitoring devices that identify sleep-disordered breathing. Even severe cases of sleep apnoea may be undiagnosed until revealed by similar technology. While treatable and perhaps worth knowing about in advance, severe sleep apnoea is also associated with higher risk for general anaesthesia and invasive procedures generally. Should patients monitored virtually at home be incentivized to collect these kinds of data, which may point towards treatable problems but also expose them to being taken off triage lists altogether due to unsuitability or higher risk for intervention? Even temperature monitoring as a means towards early identification of infection could potentially delay a given patient’s treatment, if an early signal prompts additional testing that functionally puts their wait time on pause. Put another way, we worry about the intersection of intense data collection with risk aversion generally, insofar as more data may reveal more reasons *not* to do a procedure. Conversely, safety and efficiency of surgical pathways may be enhanced through remote, digital completion of pre-assessment and proactive investigation. For example, at ICHNT, patients engage with the digital platform to complete procedural pre-assessment – confirming comorbidites, medication and social information. This also improves pathway efficiency by reducing volume and duration of pre-assessment telephone calls.

The growth of medical-grade and consumer diagnostics may tempt patients or those managing virtual wards to functionally reproduce or even go beyond a “hospital at home” like environment, but perhaps without the supervised focus on the primary cardiovascular pathology at issue. Consider further the potential for diagnostics identifying problems that are less treatable than sleep apnoea, such as cognitive impairment. This too may impact risk stratification or perceived benefits of a “P2” intervention. Even more plausibly, open-ended, open-source home-based diagnostics also raise the possibility of overwhelming the virtual ward management hub, not only with sheer volume but also with a diverse and dizzying array of diagnostics that may confound interpretation and consume scarce resources.

Potential solutions here include carefully circumscribing which diagnostics are allowable for “upload” to the virtual clinic, defined without discouraging patients and their carers from taking on the necessarily muscular role that digitally-supported triage demands, and having clear, protocolised pathways in place for actioning inbound results. For example, the remote prioritisation pathway at ICHNT operates on a rule-based escalation system. Data from ICHNT indicates that patients engage with the questionnaire for a median of six and mean ± SD of 7.1 ± 5.4 weeks. 70.8% of weekly questionnaire responses are flagged as amber or red by the automated risk matrix (see Supplementary Table 1), requiring review by non-specialist nurses in the Virtual Hospital. 33% of these are classified as having week-on-week deterioration, triggering escalation to the Cardiac specialist team.

Establishing a clear structure for measuring *population health outcomes* for the conditions of interest will inform whether the diagnostic data collected (surveys, digital data) and management algorithm is considered successful. For example, it is critically important to be certain and transparent about whether the goal of digitally-supported triage is to:

- Reduce number of cases that become emergent interventions
- Lower the overall mortality for the condition of interest
- Reduce overall waiting times and/or the length of the waitlist
- Improve the fitness of patients at the time of their procedure
- Improve patient and provider satisfaction with care
- Minimize the number of “P2” patients who die waiting for care – whether at home or in hospital
- Reduce sociodemographic disparities in outcomes

We observe that these goals are not necessarily exclusive, or clearly compatible, which is why it is so important to articulate what the programme aims to achieve clinically and to ensure that digitally-supported triage drives these goals forward. A critical determinant of goal-setting is the anticipated duration of scarcity^11^. Resource allocation in the NHS is historically anchored in treating people equally, and maximising the total benefits to society (utilitarianism)^12,13^. Spiralling waiting list times have promoted adoption of the ‘rule of rescue’ (prioritarianism), on the assumption that political and economic interventions will restore capacity in the short to medium-term. However, should this situation remain the ‘new normal’, a compound failure to consider prognosis may prove universally damaging. For example, a patient awaiting valve replacement with worsening breathlessness from aortic stenosis may experience a more complicated post-operative course, lose substantial life-years from incipient heart failure, and suffer frequent hospitalisation—which itself drives hospital bed occupancy and so reduces elective capacity.

## Summary

Digitally-supported triage represents a necessary response to an urgent clinical and population-health problem, which admirably puts the patient need first and the technology second. While we observe important concerns about the way in which this technology becomes deployed, all of this is balanced against an established and compelling counterfactual: what are the risks of *not* providing remote monitoring in this fashion? Patients are dying and suffering unplanned healthcare events whilst on waiting lists, with operating models that have thus far maintained the status quo of ad-hoc, heterogeneous or unsustainable outreach and monitoring – both between and within NHS organisations. Establishing clear programmatic goals, metrics designed to assess its success, and carefully observing the sociodemographic and clinical impact of digitally-supported triage will be essential steps in measuring the real-world impact of this groundbreaking approach to cardiovascular care.

### Supplementary information

Supplementary Material

## References

[1] Joseph P (2017). Reducing the Global Burden of Cardiovascular Disease, Part 1: The epidemiology and risk factors. Circ. Res.

[2] Knuuti J (2020). 2019 ESC Guidelines for the diagnosis and management of chronic coronary syndromes. Eur. Heart J..

[3] Vahanian A (2022). 2021 ESC/EACTS Guidelines for the management of valvular heart disease. Eur. Heart J..

[4] Mahase E (2022). Covid-19: Pandemic disruption linked to 30 000 excess heart disease deaths, charity reports. BMJ.

[5] Stickels CP (2022). Aortic stenosis post-COVID-19: A mathematical model on waiting lists and mortality. BMJ Open.

[6] Martin GP (2021). Indirect Impact of the COVID-19 pandemic on activity and outcomes of transcatheter and surgical treatment of aortic stenosis in England. Circ. Cardiovasc Inter..

[7] Anita Z (2021). Identifying ethical values for guiding triage decisions during the COVID-19 pandemic: an Italian ethical committee perspective using Delphi methodology. BMJ Open.

[8] Parker WF (2019). Association of transplant center with survival benefit among adults undergoing heart transplant in the United States. JAMA.

[9] Michie S, van Stralen MM, West R (2011). The behaviour change wheel: a new method for characterising and designing behaviour change interventions. Implement Sci..

[10] Parker WF (2020). Practice changes at U.S. transplant centers after the new adult heart allocation policy. J. Am. Coll. Cardiol..

[11] Emanuel EJ (2020). Fair allocation of scarce medical resources in the time of Covid-19. N. Engl. J. Med..

[12] Persad G, Wertheimer A, Emanuel EJ (2009). Principles for allocation of scarce medical interventions. Lancet.

[13] Persad G, Peek ME, Emanuel EJ (2020). Fairly prioritizing groups for access to COVID-19 vaccines. JAMA.

