# Supplementary material for: From ether to ethernet: ensuring ethical policy in digital transformation of waitlist triage for cardiovascular procedures
Source: NPJ Digit Med. 2024 Feb 29;7:51. doi: 10.1038/s41746-024-01019-6 (PMC10904820; doi:10.1038/s41746-024-01019-6)
Supplement: Supplementary file 1 — Supplementary Material [file 41746_2024_1019_MOESM1_ESM.pdf]

Supplementary Table 1: Risk Categorization Matrix

Cardiac Waiting List Symptom Questionnaire – red and amber responses

Chest pain after mild exertion	Chest pain after moderate exertion
Chest pain at rest	Chest pain 1-2 times in last week
Chest pain 3-4 times in last week	Chest pain slightly worse in last week
Chest pain daily or more	Chest pain stops patient after 200-500metres of walking
Chest pain moderately worse in last week	Using GTN once per week
Chest pain much worse in last week	Breathlessness the same compared to last week
Chest pain stops patient after 50-200metres of walking	Breathlessness stops patient after 200-500 metres of walking
Chest pain stops patient after <50metres of walking	Same number of pillows to sleep (at least 2)
Using GTN multiple times per day	Ankle swelling the same this week
Using GTN most/every day	Sometimes using more water tablets than usual
Breathlessness slightly worse than last week	Severe dizziness in the last month
Breathlessness much worse than last week	Dizzy spells 1-2 times in last week
Breathlessness slows/stops patient frequently	Dizziness getting worse this week
Breathlessness slows/stops patient very frequently	Palpitations 1-2x per week
Breathlessness stops patient after 50-200metres of walking	Palpitations the same this week
Breathlessness stops patient after <50metres of walking	Palpitations make patient feel unwell
Using more pillows when sleeping this week	Palpitations prolonged, with regular beat
Had to sleep upright this week	Any other symptoms with strenuous activity
Ankle swelling slightly worse this week	

Ankle swelling much worse this week	
Using consistently more water tablets than usual	
Fainted in last month	
Nearly fainted in the last month	
Dizzy spells 3-4 times in last week	
Dizzy spells daily or more	
Dizziness much worse this week	
Dizziness at rest	
Dizziness on exertion	
Palpitations daily	
Palpitations 3-4x per week	
Palpitations slightly worse this week	
Palpitations much worse this week	
Palpitations cause severe breathlessness, near fainting or fainting	
Palpitations make patient feel very unwell	
Palpitations prolonged, with irregular beat	
Palpitations continuous	
Any other symptoms on mild exertion	
Any other symptoms at rest	

Red Patients Identified patients should be reviewed by the NWL Virtual Hospital team within 1 working day & if there are new abnormal or worsening symptoms, escalated to the Cardiac Preassessment team. The Cardiac Preassessment team will escalate to the Surgical Fellow or Consultant as clinically appropriate.

- Patients reporting chest pain at REST or fainting should be escalated to the Cardiac Preassessment team

Amber Patients Identified patients should be reviewed by the NWL Virtual Hospital team within 2 working days & if there are new abnormal or worsening symptoms, escalated to the Cardiac Preassessment team. The Cardiac Preassessment team will escalate to the Surgical Fellow or Consultant as clinically appropriate.

Supplementary Figure 1: Prioritization Processes Incorporating Survey Responses and Alerts for Clinical Changes Requiring Intervention

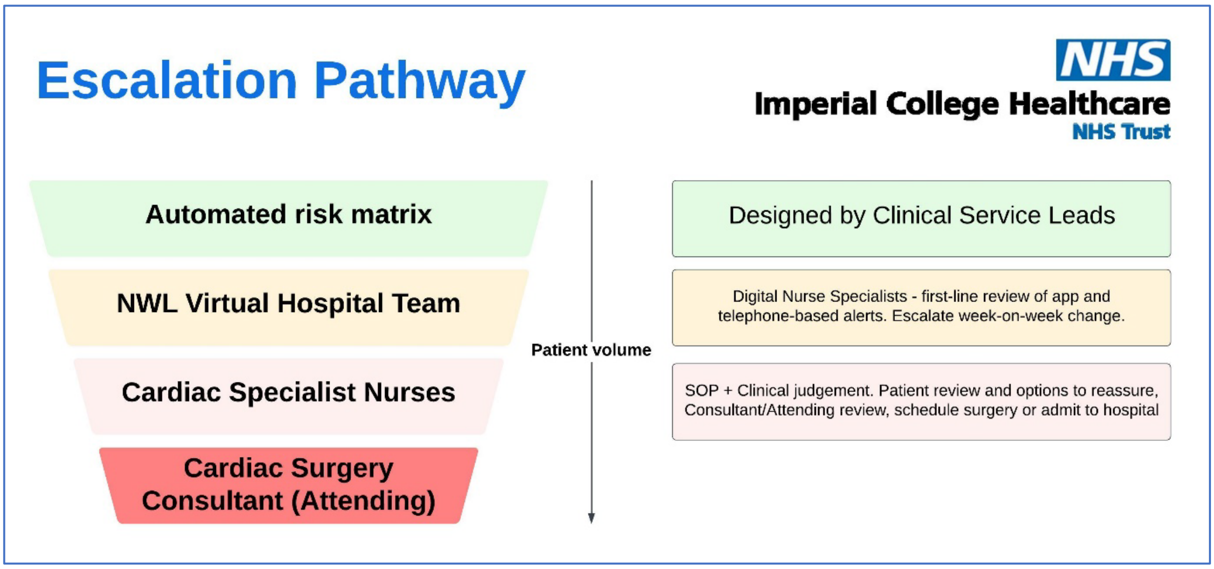

Supplementary Note 1: Symptom Assessment Ortus Platform Questions

DOMAIN 1

Chest Pain

Screening question 1: **In the last week, have you experienced Chest pain / ache / tightness / heaviness – if yes, proceed to detailed questions below; if not, proceed to next domain**

1. **In the last week**, have you experienced Chest pain/ache/tightness/heaviness after exertion: (severity)

a) After heavy exertion (green)	
b) After moderate exertion (amber)	
c) After mild exertion (red)	
d) At rest (red)	

2. How often do you get chest pain/ache/tightness or heaviness: (frequency)

a) None in the last week (green)	
b) 1-2 times in the last week (amber)	
c) 3-4 times in the last week (red)	
d) Daily or more (red)	

3. Has this chest pain/ache/tightness or heaviness changed in the last WEEK? (progression)

a) Unchanged (green)	
b) Slightly worse (amber)	
c) Moderately worse (red)	
d) Much worse (red)	

4. How far are you able to walk before chest pain stops you?

a) Unlimited (500 metres +) (green)	
b) 200-500 metres (amber)	
c) 50-200 metres (red)	
d) <50 metres (red)	

5. Have you used a GTN spray or tablets in the last week? (need for treatment)

a) No (green)	
b) Once a week (amber)	
c) Multiple times a day (red)	
d) Most/every day(s) (red)	

<u>DOMAIN 2</u>

<u>Breathlessness/HF</u>

<u>Screening question 2</u>: In the last week, have you experienced undue breathlessness or ankle swelling? If no, proceed to next domain

6. Over the last week, has your breathlessness been: (progression)

a) Absent (green)	
b) The same (amber)	
c) Slightly worse (red)	
d) Much worse (red)	

7. Does your breathlessness: (severity)

a) Not slow you down (green)	
b) Slow you down a little (green)	
c) Slow you/stop you frequently (red)	
d) Slow you/stop you very frequently (red)	

8. How far can you walk on the flat before breathlessness stops you?

a) Unlimited (500 metres +) (green)	
b) 200-500 metres (amber)	
c) 50-200 metres (red)	
d) <50 metres (red)	

9. Have you been using extra pillows when sleeping in the last week?

a) No extra pillows (green)	
b) Same as usual (with at least 2 pillows) (amber)	
c) Needed more pillows (red)	
d) Had to sit upright (red)	

10. Have you experienced swollen ankles in the last week?

a) Not swollen (green)	
b) The same (amber)	
c) Slightly worse (red)	
d) Much worse (red)	

11. Has there been a change in water tablet daily dose in the last week:

a) Not on any (green)	
b) The same dose as usual (green)	
c) Sometimes more than usual (amber)	
d) Consistently more than usual (red)	

DOMAIN 3

Syncope/Fainting

Screening question 3: In the last **MONTH**, have you experienced any unexpected dizziness, blackouts, near fainting or fainting? If no, proceed to next domain

12. How bad were these symptoms? (Severity)

a) Mild dizziness (green)	
b) Severe dizziness (amber)	
c) Near fainting (red)	
d) Fainting (red)	

13. How frequent were any dizzy spells? (Frequency)

a) None in the last week (green)	
b) 1-2 times in the last week (amber)	
c) 3-4 times in the last week (red)	
d) Daily or more (red)	

14. Has the dizziness been: (progression)

a) Absent (green)	
b) Mild/stable/short lived (green)	
c) Getting worse (amber)	
d) Much worse (red)	

15. If you have had serious dizziness / near fainting / fainting was this: (circumstances)

a) Absent (green)	
b) When getting up or bending over (green)	
c) At rest (red)	
d) On exertion (red)	

Domain 4

Palpitations If answer is no move to next domain

Screening question 4: In the last week, have you experienced any palpitations lasting for more than 2-3 seconds?

16. In the last week, how frequently have you had palpitations? (frequency)

a) Not at all (green)	
b) 1-2 week (amber)	
c) 3-4 week (red)	
d) Daily (red)	

17. In the last week, have your palpitations been: (progression)

a) Absent (green)	
b) The same (amber)	
c) Slightly worse (red)	
d) Much worse (red)	

18. Do your palpitations (severity)

a) Cause minimal concern (green)	
b) Make you feel unwell (amber)	
c) Make you feel very unwell (red)	
d) Cause severe breathlessness, near fainting or fainting (red)	

19. Are your palpitations

a) Momentary or a few seconds long (green)	
b) Prolonged, with regular beat (amber)	
c) Prolonged, with irregular beat (red)	
d) Continuous (regular or irregular beat) (red)	

Any other symptoms

20. If you had any other symptoms {insert text} was it:

a) The same (green)	
b) With strenuous activity (amber)	
c) On mild exertion (red)	
d) At rest (red)	
